# Limitations of Bayesian Leave-One-Out Cross-Validation for Model Selection

**DOI:** 10.1007/s42113-018-0011-7

**Published:** 2018-09-27

**Authors:** Quentin F. Gronau, Eric-Jan Wagenmakers

**Affiliations:** University of Amsterdam, Amsterdam, Netherlands

**Keywords:** Generalizability, Consistency, Evidence, Bounded support, Induction, Principle of parsimony

## Abstract

Cross-validation (CV) is increasingly popular as a generic method to adjudicate between mathematical models of cognition and behavior. In order to measure model generalizability, CV quantifies out-of-sample predictive performance, and the CV preference goes to the model that predicted the out-of-sample data best. The advantages of CV include theoretic simplicity and practical feasibility. Despite its prominence, however, the limitations of CV are often underappreciated. Here, we demonstrate the limitations of a particular form of CV—Bayesian leave-one-out cross-validation or LOO—with three concrete examples. In each example, a data set of infinite size is perfectly in line with the predictions of a simple model (i.e., a general law or invariance). Nevertheless, LOO shows bounded and relatively modest support for the simple model. We conclude that CV is not a panacea for model selection.

[...] if you can’t do simple problems, how can you do complicated ones? Dennis Lindley (1985, p. 65)

Model selection is a perennial problem, both in mathematical psychology (e.g., the three special issues for the *Journal of Mathematical Psychology*: Mulder and Wagenmakers 2016; Myung et al. 2000; Wagenmakers and Waldorp 2006) and in statistics (e.g., Ando 2010; Burnham and Anderson 2002; Claeskens and Hjort 2008; Grünwald et al. 2005; Wrinch and Jeffreys 1921). The main challenge for model selection is known both as the bias-variance tradeoff and as the parsimony-fit tradeoff (e.g., Myung and Pitt 1997; Myung 2000). These tradeoffs form the basis of what may be called the *fundamental law of model selection*: when the goal is to assess a model’s predictive performance, goodness-of-fit ought to be discounted by model complexity. For instance, consider the comparison between two regression models, $\mathcal {M}_{S}$ and $\mathcal {M}_{C}$; the “simple” model $\mathcal {M}_{S}$ has *k* predictors, whereas the “complex” model $\mathcal {M}_{C}$ has *l* predictors more, for a total of $k+l$. Hence, $\mathcal {M}_{S}$ is said to be nested under $\mathcal {M}_{C}$. In such cases, $\mathcal {M}_{C}$ always outperforms $\mathcal {M}_{S}$ in terms of goodness-of-fit (e.g., variance explained), even when the *l* extra predictors are useless in the sense that they capture only the idiosyncratic, nonreplicable noise in the sample at hand. Consequently, model selection methods that violate the fundamental law trivially fail, because they prefer the most complex model regardless of the data.

All popular methods of model selection adhere to the fundamental law in that they seek to chart a route that avoids the Scylla of “overfitting” (i.e., overweighting goodness-of-fit such that complex models receive an undue preference) and the Charybdis of “underfitting” (i.e., overweighting parsimony such that simple models receive an undue preference). Both Scylla and Charybdis result in the selection of models with poor predictive performance; models that fall prey to Scylla mistake what is idiosyncratic noise in the sample for replicable signal, leading to excess variability in the parameter estimates; in contrast, models that fall prey to Charybdis mistake what is replicable signal for idiosyncratic noise, leading to bias in the parameter estimates. Both excess variability and bias result in suboptimal predictions, that is, poor generalizability.

The cornucopia of model selection methods includes (1) approximate methods such as AIC (Akaike 1973) and BIC (Nathoo and Masson 2016; Schwarz 1978), which punish complexity by an additive term that includes the number of free parameters; (2) methods that quantify predictive performance by averaging goodness-of-fit across the model’s entire parameter space (i.e., the Bayes factor, e.g., Jeffreys 1961; Kass and Raftery 1995; Ly et al. 2016; Rouder et al. 2012); note that the averaging process indirectly penalizes complexity, as a vast parameter space will generally contain large swathes that produce a poor fit (Vandekerckhove et al. 2015); (3) methods based on minimum description length (Grünwald 2007; Myung et al. 2006; Rissanen 2007), where the goal is the efficient transmission of information, that is, a model and the data it encodes; complex models take more bits to describe and transmit; and (4) methods such as cross-validation (CV; Browne 2000; Stone 1974) that assess predictive performance directly, namely by separating the data in a part that is used for fitting (i.e., the calibration set or training set) and a part that is used to assess predictive adequacy (i.e., the validation set or test set).

Each model selection method comes with its own set of assumptions and operating characteristics which may or may not be appropriate for the application at hand. For instance, AIC and BIC assume that model complexity can be approximated by counting the number of free parameters, and the Bayes factor presupposes the availability of a reasonable joint prior distribution across the parameter space (Lee and Vanpaemel 2018). The focus of the current manuscript is on CV, an increasingly popular and generic model selection procedure (e.g., Doxas et al. 2010; Hastie et al. 2008; Yarkoni and Westfall 2017). Specifically, our investigation concerns leave-one-out CV, where the model is trained on all observations except one, which then forms the test set. The procedure is repeated for all *n* observations, and the overall predictive CV performance is the sum of the predictive scores for each of the *n* test sets.

Originally developed within a frequentist framework, leave-one-out CV can also be executed within a Bayesian framework; in the Bayesian framework, the predictions for the test sets are based not on a point estimate but on the entire posterior distribution (Geisser and Eddy 1979; Gelfand et al. 1992; see also Geisser 1975). Henceforth, we will refer to this Bayesian version of leave-one-out CV as LOO (e.g., Gelman et al. 2014; Vehtari and Ojanen 2012; Vehtari et al.^2017^).^1^

To foreshadow our conclusion, we demonstrate below with three concrete examples how LOO can yield conclusions that appear undesirable; specifically, in the idealized case where there exists a data set of infinite size that is perfectly consistent with the simple model $\mathcal {M}_{S}$, LOO will nevertheless fail to strongly endorse $\mathcal {M}_{S}$. It has long been known that CV has this property, termed “inconsistency” (e.g., Shao^1993^).^2^ Our examples demonstrate not just that CV is inconsistent, but also serve to explicate the reason for the inconsistency. Moreover, the examples show not only that CV is inconsistent, that is, the support for the true $\mathcal {M}_{S}$ does not increase without bound,^3^ but they also show that the degree of the support for the true $\mathcal {M}_{S}$ is surprisingly modest. One of our examples also reveals that, in contrast to what is commonly assumed, the results for LOO can depend strongly on the prior distribution, even asymptotically; finally, in all three examples, the observation of data perfectly consistent with $\mathcal {M}_{S}$ may nevertheless cause LOO to decrease its preference for $\mathcal {M}_{S}$. Before we turn to the three examples, we first introduce LOO in more detail.

## Bayesian Leave-One-Out Cross-Validation

The general principle of cross-validation is to partition a data set consisting of *n* observations $y_{1}, y_{2}, \ldots , y_{n}$ into a training set and a test set. The training set is used to fit the model and the test set is used to evaluate the fitted model’s predictive adequacy. LOO repeatedly partitions the data set into a training set which consists of all data points except the *i* th one, denoted as $y_{-i}$, and then evaluates the predictive density for the held-out data point $y_{i}$. The log of these predictive densities for all data points is summed to obtain the LOO estimate of the expected log pointwise predictive density (elpd; Gelman et al. 2014; Vehtari et al.^2017^):^4^

1 $$ \text{elpd}_{\text{loo}} = \sum\limits_{i = 1}^{n} \log p(y_{i} \mid y_{-i}), $$

where

2 $$ p(y_{i} \mid y_{-i}) = \int p(y_{i} \mid \theta) p(\theta \mid y_{-i}) \text{d}\theta $$

is the leave-one-out predictive density for data point $y_{i}$ given the remaining data points $y_{-i}$ and $\theta $ denotes the model parameters.

It is insightful to note the close connection of LOO to what Gelfand and Dey (1994) called the *pseudo-Bayes factor* (PSBF) which they attribute to Geisser and Eddy (1979). Recall that the Bayes factor that compares models $\mathcal {M}_{1}$ and $\mathcal {M}_{2}$ (Kass and Raftery 1995) is defined as:

3 $$ \text{BF}_{12} = \frac{p(y \mid \mathcal{M}_{1})}{p(y \mid \mathcal{M}_{2})}, $$

where $y = (y_{1}, y_{2}, \ldots , y_{n})$ and $p(y \mid \mathcal {M}_{m}) = {\int }_{{\Theta }_{m}} p(y \mid \theta _{m}, \mathcal {M}_{m}) p(\theta _{m} \mid \mathcal {M}_{m}) \text {d}\theta _{m}$ denotes the marginal likelihood of model $\mathcal {M}_{m}$, $m \in \{1,2\}$. The pseudo-Bayes factor (PSBF) replaces the marginal likelihood of each model by the product of the leave-one-out predictive densities so that:

4 $$\begin{array}{@{}rcl@{}} \text{PSBF}_{12} &=& \frac{\displaystyle{\prod}_{i = 1}^{n} p(y_{i} \mid y_{-i}, \mathcal{M}_{1})}{\displaystyle{\prod}_{i = 1}^{n} p(y_{i} \mid y_{-i}, \mathcal{M}_{2})} \\ &=& \exp\left\{{\Delta}\text{elpd}_{\text{loo}}^{\mathcal{M}_{1}, \mathcal{M}_{2}}\right\}, \end{array} $$

where ${\Delta }\text {elpd}_{\text {loo}}^{\mathcal {M}_{1}, \mathcal {M}_{2}} = \text {elpd}_{\text {loo}}^{\mathcal {M}_{1}} - \text {elpd}_{\text {loo}}^{\mathcal {M}_{2}} $ and $\text {elpd}_{\text {loo}}^{\mathcal {M}_{m}}$ denotes the LOO estimate for model $\mathcal {M}_{m}$, $m \in \{1,2\}$. It is also worth mentioning that LOO can be used to compute model weights (e.g., Yao et al. in press; see also Burnham and Anderson 2002; Wagenmakers and Farrell 2004) as follows:

5 $$ w_{m} = \frac{\exp\left\{\text{elpd}_{\text{loo}}^{\mathcal{M}_{m}}\right\}}{\displaystyle{\sum}_{j = 1}^{M} \exp\left\{\text{elpd}_{\text{loo}}^{\mathcal{M}_{j}}\right\}}, $$

where $w_{m}$ denotes the model weight for model $\mathcal {M}_{m}$ and *M* is the number of models under consideration. The LOO results from the three examples below will be primarily presented as weights.

## Example 1: Induction

As a first example, we consider what is perhaps the world’s oldest inference problem, one that has occupied philosophers for over two millennia: given a general law such as “all *X*’s have property *Y*,” how does the accumulation of confirmatory instances (i.e., *X*’s that indeed have property *Y* ) increase our confidence in the general law? Examples of such general laws include “all ravens are black,” “all apples grow on apple trees,” “all neutral atoms have the same number of protons and electrons,” and “all children with Down syndrome have all or part of a third copy of chromosome 21.”

To address this question statistically, we can compare two models (e.g., Etz and Wagenmakers 2017; Wrinch and Jeffreys 1921). The first model corresponds to the general law and can be conceptualized as $\mathcal {H}_{0}: \theta = 1$, where $\theta $ is a Bernoulli probability parameter. This model predicts that only confirmatory instances are encountered. The second model relaxes the general law and is therefore more complex; it assigns $\theta $ a prior distribution, which, for mathematical convenience, we take to be from the beta family— consequently, we have $\mathcal {H}_{1}: \theta \sim \text {Beta}(a,b)$.

In the following, we assume that, in line with the prediction from $\mathcal {H}_{0}$, only confirmatory instances are observed. In such a scenario, we submit that there are at least three desiderata for model selection. First, for any sample size $n>0$ of confirmatory instances, the data ought to support the general law $\mathcal {H}_{0}$; second, as *n* increases, so should the level of support in favor of $\mathcal {H}_{0}$; third, as *n* increases without bound, the support in favor of $\mathcal {H}_{0}$ should grow infinitely large.

How does LOO perform in this scenario? Before proceeding, note that when LOO makes predictions based on the maximum likelihood estimate (MLE), none of the above desiderata are fulfilled. Any training set of size $n-1$ will contain $k=n-1$ confirmatory instances, such that the MLE under $\mathcal {H}_{1}$ is $\hat {\theta }=k/(n-1)= 1$; of course, the general law $\mathcal {H}_{0}$ does not contain any adjustable parameters and simply stipulates that $\theta = 1$. When the models’ predictive performance is evaluated for the test set observation, it then transpires that both $\mathcal {H}_{0}$ and $\mathcal {H}_{1}$ have $\theta $ set to 1 ($\mathcal {H}_{0}$ on principle, $\mathcal {H}_{1}$ by virtue of having seen the $n-1$ confirmatory instances from the training set), so that they make identical predictions. Consequently, according to the maximum likelihood version of LOO, the data are completely uninformative, no matter how many confirmatory instances are observed.^5^

The Bayesian LOO makes predictions using the leave-one-out posterior distribution for $\theta $ under $\mathcal {H}_{1}$, and this means that it at least fulfills the first desideratum: the prediction under $\mathcal {H}_{0}: \theta = 1$ is perfect, whereas the prediction under $\mathcal {H}_{1}: \theta \sim \text {Beta}(a+n-1,b)$ involves values of $\theta $ that do not make such perfect predictions. As a result, the Bayesian LOO will show that the general law $\mathcal {H}_{0}$ outpredicts $\mathcal {H}_{1}$ for the test set.

What happens when sample size *n* grows large? Intuitively, two forces are in opposition: on the one hand, as *n* grows large, the leave-one-out posterior distribution of $\theta $ under the complex model $\mathcal {H}_{1}$ will be increasingly concentrated near 1, generating predictions for the test set data that are increasingly similar to those made by $\mathcal {H}_{0}$. On the other hand, even with *n* large, the predictions from $\mathcal {H}_{1}$ will still be inferior to those from $\mathcal {H}_{0}$, and these inferior predictions are multiplied by *n*, the number of test sets.

As it turns out, these two forces are asymptotically in balance, so that the level of support in favor of $\mathcal {H}_{0}$ approaches a bound as *n* grows large. We first provide the mathematical result and then show the outcome for a few select scenarios.

### Mathematical Result

In example 1, the data consist of *n* realizations drawn from a Bernoulli distribution, denoted by $y_{i}$, $i = 1,2,\ldots , n$. Under $\mathcal {H}_{0}$, the success probability *𝜃* is fixed to 1 and under $\mathcal {H}_{1}$, $\theta $ is assigned a $\text {Beta}(a, b)$ prior. We consider the case where only successes are observed, that is, *y*_*i*_ = 1,∀*i* ∈{1,2,…, *n*}. The model corresponding to $\mathcal {H}_{0}: \theta = 1$ has no free parameters and predicts $y_{i} = 1$ with probability one. Therefore, the Bayesian LOO estimate $\text {elpd}_{\text {loo}}^{\mathcal {H}_{0}}$ is equal to 0. To calculate the LOO estimate under $\mathcal {H}_{1}$, one needs to be able to evaluate the predictive density for a single data point given the remaining data points. Recall that the posterior based on $n-1$ observations is a $\text {Beta}(a + n - 1, b)$ distribution. Consequently, the leave-one-out predictive density is obtained as a generalization (with *a* and *b* potentially different from 1) of Laplace’s rule of succession applied to $n-1$ observations,

6 $$\begin{array}{@{}rcl@{}} p(y_{i} \mid y_{-i}) &=& {{\int}_{0}^{1}} \underbrace{\theta}_{p(y_{i} \mid \theta)} \underbrace{\tfrac{{\Gamma}\left( a + n - 1 + b\right)}{{\Gamma}\left( a + n - 1\right) {\Gamma}(b)} \theta^{a + n - 2} \left( 1 - \theta\right)^{b - 1}}_{p(\theta \mid y_{-i})} \text{d}\theta \\ &=& \frac{a + n - 1}{a + n - 1 + b}, \end{array} $$

and the Bayesian LOO estimate under $\mathcal {H}_{1}$ is given by

7 $$ \text{elpd}_{\text{loo}}^{\mathcal{H}_{1}} = n \log\left( \frac{a + n - 1}{a + n - 1 + b}\right). $$

The difference in the LOO estimates is

8 $$\begin{array}{@{}rcl@{}} {\Delta}\text{elpd}_{\text{loo}}^{\mathcal{H}_{0}, \mathcal{H}_{1}} &=& \text{elpd}_{\text{loo}}^{\mathcal{H}_{0}} - \text{elpd}_{\text{loo}}^{\mathcal{H}_{1}} \\ &=& - n \log\left( \frac{a + n - 1}{a + n - 1 + b}\right). \end{array} $$

As the number of confirmatory instances *n* grows large, the difference in the LOO estimates approaches a bound (see Appendix A for a derivation):

9 $$ \lim_{n \to \infty} {\Delta}\text{elpd}_{\text{loo}}^{\mathcal{H}_{0}, \mathcal{H}_{1}} = b. $$

Hence, the asymptotic difference in the Bayesian LOO estimates under $\mathcal {H}_{0}$ and under $\mathcal {H}_{1}$ equals the Beta prior parameter *b*. Consequently, the limit of the pseudo-Bayes factor is

10 $$ \lim_{n \to \infty} \text{PSBF}_{01} = \exp\left\{b\right\}, $$

and the limit of the model weight for $\mathcal {H}_{0}$ is

11 $$ \lim_{n \to \infty} w_{0} = \frac{\exp\left\{b\right\}}{1 + \exp\left\{b\right\}}.  $$

### Select Scenarios

**Fig. 1:**
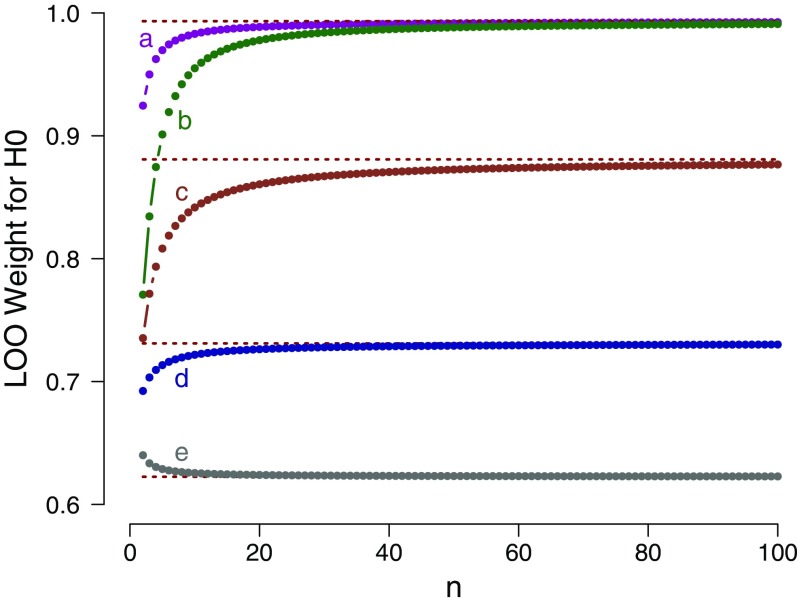
Example 1: LOO weights for $\mathcal {H}_{0}: \theta = 1$ as a function of the number of confirmatory instances *n*, evaluated in relation to five different prior specifications for $\mathcal {H}_{1}$: **a** $\mathcal {H}_{1}: \theta \sim \text {Beta}(1, 5)$; **b** $\mathcal {H}_{1}: \theta \sim \text {Beta}(5, 5)$; **c** $\mathcal {H}_{1}: \theta \sim \text {Beta}(2, 2)$; **d** $\mathcal {H}_{1}: \theta \sim \text {Beta}(1, 1)$; and **e** $\mathcal {H}_{1}: \theta \sim \text {Beta}(0.5, 0.5)$. The dotted horizontal lines indicate the corresponding analytical asymptotic bounds (see text for details). Available at https://tinyurl.com/ya2r4gx8 under CC license https://creativecommons.org/licenses/by/2.0/

The mathematical result can be applied to a series of select scenarios. Figure 1 shows the LOO weight in favor of the general law $\mathcal {H}_{0}$ as a function of the number of confirmatory instances *n*, separately for five different prior specifications under $\mathcal {H}_{1}$. The figure confirms that for each prior specification, the LOO weight for $\mathcal {H}_{0}$ approaches its asymptotic bound as *n* grows large.

We conclude the following: (1) as *n* grows large, the support for the general law $\mathcal {H}_{0}$ approaches a bound; (2) for many common prior distributions, this bound is surprisingly low. For instance, the Laplace prior $\theta \sim \text {Beta(1,1)}$ (case **d**) yields a weight of $e/(1 + e) \approx 0.731$; (3) contrary to popular belief, our results provide an example of a situation in which the results from LOO are highly dependent on the prior distribution, even asymptotically. This is clear from Eq. 11 and evidenced in Fig. 1; and (4) as shown by case **e** in Fig. 1, the choice of Jeffreys’s prior (i.e., $\theta \sim \text {Beta}(0.5,0.5)$) results in a function that approaches the asymptote from above. This means that, according to LOO, the observation of additional confirmatory instances actually decreases the support for the general law, violating the second desideratum outlined above. This violation can be explained by the fact that the confirmatory instances help the complex model $\mathcal {H}_{1}$ concentrate more mass near 1, thereby better mimicking the predictions from the simple model $\mathcal {H}_{0}$. For some prior choices, this increased ability to mimic outweighs the fact that the additional confirmatory instances are better predicted by $\mathcal {H}_{0}$ than by $\mathcal {H}_{1}$.

One counterargument to this demonstration could be that, despite its venerable history, the case of induction is somewhat idiosyncratic, having to do more with logic than with statistics. To rebut this argument, we present two additional examples.

## Example 2: Chance

As a second example, we consider the case where the general law states that the Bernoulli probability parameter $\theta $ equals $1/2$ rather than 1. Processes that may be guided by such a law include “the probability that a randomly chosen digit from the decimal expansion of $\pi $ is odd rather than even” (Gronau and Wagenmakers in press), “the probability that a particular uranium-238 atom will decay in the next 4.5 billion years,” or “the probability that an extrovert participant in an experiment on extra-sensory perception correctly predicts whether an erotic picture will appear on the right or on the left side of a computer screen” (Bem 2011).

Hence, the general law holds that $\mathcal {H}_{0}: \theta = 1/2$, and the model that relaxes that law is given by $\mathcal {H}_{1}: \theta \sim \text {Beta}(a,b)$, as in example 1. Also, similar to example 1, we consider the situation where the observed data are perfectly consistent with the predictions from $\mathcal {H}_{0}$. To accomplish this, we consider only even sample sizes *n* and set the number of successes *k* equal to $n/2$. In other words, the binary data come as pairs, where one member is a success and the other is a failure. The general desiderata are similar to those from example 1: First, for any sample size with $k=n/2$ successes, the data ought to support the general law $\mathcal {H}_{0}$; second, as *n* increases (for *n* even and with $k=n/2$ successes), so should the level of support in favor of $\mathcal {H}_{0}$; third, as *n* increases without bound, the support in favor of $\mathcal {H}_{0}$ should grow infinity large.

### Mathematical Result

In example 2, the data consist again of *n* realizations drawn from a Bernoulli distribution, denoted by $y_{i}$, $i = 1,2,\ldots , n$. Under $\mathcal {H}_{0}$, the success probability *𝜃* is now fixed to $1/2$; under $\mathcal {H}_{1}$, $\theta $ is again assigned a $\text {Beta}(a, b)$ prior. The model corresponding to $\mathcal {H}_{0}: \theta = 1/2$ has no free parameters and predicts $y_{i} = 0$ with probability $1/2$ and $y_{i} = 1$ with probability $1/2$. Therefore, the LOO estimate is given by $\text {elpd}_{\text {loo}}^{\mathcal {H}_{0}} = -n \log \left (2\right )$. To calculate the LOO estimate under $\mathcal {H}_{1}$, one needs to be able to evaluate the predictive density for a single data point given the remaining data points. Recall that the posterior based on $n-1$ observations is a $\text {Beta}(a + k_{-i}, b + n - 1 - k_{-i})$ distribution, where $k_{-i} = {\sum }_{j \ne i} y_{j}$ denotes the number of successes based on all data points except the *i* th one. Consequently, the leave-one-out predictive density is given by:

12 $$\begin{array}{@{}rcl@{}} p(y_{i} \!\mid\! y_{-i}) \!\!&=&\!\! {{\int}_{0}^{1}}\! \underbrace{\theta^{y_{i}} (1 - \theta)^{1 - y_{i}}}_{p(y_{i} \mid \theta)}\\ &&\times \underbrace{\tfrac{{\Gamma}\left( a + b + n - 1\right)}{{\Gamma}\left( a + k_{-i}\right) {\Gamma}(b + n- k_{-i} - 1)} \theta^{a + k_{-i} - 1} \!\left( 1 - \theta\right)^{b + n - k_{-i} - 2}}_{p(\theta \mid y_{-i})} \text{d}\theta \\ &=& \left\{\begin{array}{ll} \frac{a + k - 1}{a + b + n - 1} & \text{if } y_{i} = 1 \\ \frac{b + n - k - 1}{a + b + n - 1} & \text{if } y_{i} = 0, \end{array}\right. \end{array} $$

where $k = {\sum }_{i = 1}^{n} y_{i}$ denotes the total number of successes. Example 2 considers the case where *n* is even and the number of successes *k* equals $\frac {n}{2}$. The Bayesian LOO estimate under $\mathcal {H}_{1}$ is then given by:

13 $$ \text{elpd}_{\text{loo}}^{\mathcal{H}_{1}} = \frac{n}{2} \log\left( \frac{a + \frac{n}{2} - 1}{a + b + n - 1}\right) + \frac{n}{2} \log\left( \frac{b + \frac{n}{2} - 1}{a + b + n - 1}\right). $$

The difference in the LOO estimates can be written as

14 $$\begin{array}{@{}rcl@{}} {\Delta}\text{elpd}_{\text{loo}}^{\mathcal{H}_{0}, \mathcal{H}_{1}} &=& \frac{n}{2} \log\left( \frac{a + b + n - 1}{2a + n - 2}\right)\\ &&+ \frac{n}{2} \log\left( \frac{a + b + n - 1}{2b + n - 2}\right). \end{array} $$

As the even sample size *n* grows large, the difference in the LOO estimates approaches a bound (see Appendix B for a derivation):

15 $$ \lim_{n \to \infty} {\Delta}\text{elpd}_{\text{loo}}^{\mathcal{H}_{0}, \mathcal{H}_{1}} = 1. $$

Consequently, the limit of the pseudo-Bayes factor is

16 $$ \lim_{n \to \infty} \text{PSBF}_{01} = e \approx 2.718, $$

and the limit of the model weight for $\mathcal {H}_{0}$ is

17 $$ \lim_{n \to \infty} w_{0} = \frac{e}{1 + e} \approx 0.731.  $$

### Select Scenarios

**Fig. 2:**
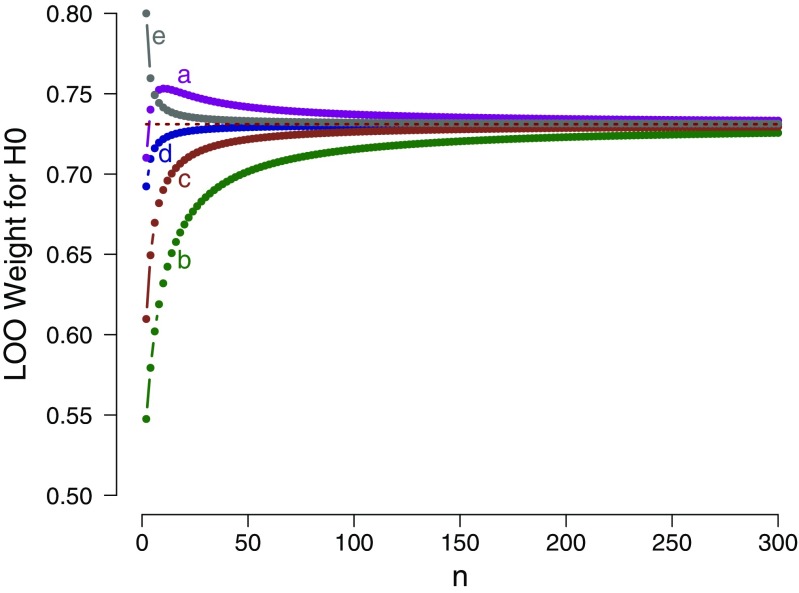
Example 2: LOO weights for $\mathcal {H}_{0}: \theta = 1/2$ as a function of the number of observations *n*, where the number of successes *k* = *n*/2, evaluated in relation to five different prior specifications for $\mathcal {H}_{1}$: **a** $\mathcal {H}_{1}: \theta \sim \text {Beta}(1, 5)$; **b** $\mathcal {H}_{1}: \theta \sim \text {Beta}(5, 5)$; **c** $\mathcal {H}_{1}: \theta \sim \text {Beta}(2, 2)$; **d** $\mathcal {H}_{1}: \theta \sim \text {Beta}(1, 1)$; and **e** $\mathcal {H}_{1}: \theta \sim \text {Beta}(0.5, 0.5)$. The dotted horizontal line indicates the corresponding analytical asymptotic bound. Note that only even sample sizes are displayed (see text for details). Available at https://tinyurl.com/y8azu4hc under CC license https://creativecommons.org/licenses/by/2.0/

The mathematical result can be applied to a series of select scenarios, as before. Figure 2 shows the LOO weight in favor of the general law $\mathcal {H}_{0}$ as a function of the even number of observations *n*, separately for five different prior specifications under $\mathcal {H}_{1}$. The figure confirms that for each prior specification, the LOO weight for $\mathcal {H}_{0}$ approaches its asymptotic bound as *n* grows large.

We conclude the following: (1) as *n* grows large, the support for the general law $\mathcal {H}_{0}$ approaches a bound; (2) in contrast to example 1, this bound is independent of the particular choice of Beta prior distribution for $\theta $ under $\mathcal {H}_{1}$; however, consistent with example 1, this bound is surprisingly low. Even with an infinite number of observations, exactly half of which are successes and half of which are failures, the model weight for the general law $\mathcal {H}_{0}$ does not exceed a modest 0.731; (3) as shown by case **e** in Fig. 2, the choice of Jeffreys’s prior (i.e., $\theta \sim \text {Beta}(0.5,0.5)$) results in a function that approaches the asymptote from above. This means that, according to LOO, the observation of additional success-failure pairs actually decreases the support for the general law, violating the second desideratum outlined above; (4) as shown by case **a** in Fig. 2, the choice of a $\text {Beta}(1,5)$ prior results in a nonmonotonic relation, where the addition of $\mathcal {H}_{0}$-consistent pairs initially increases the support for $\mathcal {H}_{0}$, and later decreases it.

In sum, the result of the LOO procedure for a test against a chance process, $\mathcal {H}_{0}: \theta = 1/2$, reveals behavior that is broadly similar to that for the test of induction ($\mathcal {H}_{0}: \theta = 0$ or $\mathcal {H}_{0}: \theta = 1$), and that violates two seemingly uncontroversial desiderata, namely that the additional observation of data that are perfectly consistent with the general law $\mathcal {H}_{0}$ ought to result in more support for $\mathcal {H}_{0}$, and do so without bound as *n* grows indefinitely. The final example concerns continuous data.

## Example 3: Nullity of a Normal Mean

As a final example, we consider the case of the *z* test: data are normally distributed with unknown mean $\mu $ and known variance $\sigma ^{2}= 1$. For concreteness, we consider a general law which states that the mean $\mu $ equals 0, that is, $\mathcal {H}_{0}: \mu = 0$. The model that relaxes the general law assigns a prior distribution to $\mu $; specifically, we consider $\mathcal {H}_{1}: \mu \sim \mathcal {N}(0,{\sigma _{0}^{2}})$. Similar to examples 1 and 2, we consider the situation where the observed data are perfectly consistent with the predictions from $\mathcal {H}_{0}$. Consequently, we consider data for which the sample mean $\bar {y}$ is exactly 0 and the sample variance $s^{2} = \frac {1}{n - 1} {\sum }_{i = 1}^{n} (y_{i} - \bar {y})^{2}$ is exactly 1.

The general desiderata are similar to those from examples 1 and 2: First, for any sample size *n* with sample mean equal to zero and sample variance equal to 1, the data ought to support the general law $\mathcal {H}_{0}$; second, as *n* increases, so should the level of support in favor of $\mathcal {H}_{0}$; third, as *n* increases without bound, the support in favor of $\mathcal {H}_{0}$ should grow infinitely large.

### Mathematical Result

In example 3, the data consist of *n* realizations drawn from a normal distribution with mean $\mu $ and known variance $\sigma ^{2} = 1$: $y_{i} \sim \mathcal {N}(\mu , 1)$, *i* = 1,2,…, *n*. Under $\mathcal {H}_{0}$, the mean $\mu $ is fixed to 0; under $\mathcal {H}_{1}$, $\mu $ is assigned a $\mathcal {N}(0, {\sigma _{0}^{2}})$ prior. The model corresponding to $\mathcal {H}_{0}: \mu = 0$ has no free parameters so that the Bayesian LOO estimate is obtained by summing the log likelihood values:

18 $$ \text{elpd}_{\text{loo}}^{\mathcal{H}_{0}} = -\frac{n}{2} \log\left( 2 \pi \right) -\frac{n - 1}{2}. $$

To calculate the LOO estimate under $\mathcal {H}_{1}$, one needs to be able to evaluate the predictive density for a single data point given the remaining data points. Recall that the posterior for $\mu $ based on $n-1$ observations is a $\mathcal {N}(\mu _{-i}, \sigma _{-i}^{2})$ normal distribution distribution, with

19 $$ \mu_{-i} = \frac{(n - 1) \bar{y}_{-i}}{n - 1 + \frac{1}{{\sigma_{0}^{2}}}}, $$

and

20 $$ \sigma_{-i}^{2} = \frac{1}{n - 1 + \frac{1}{{\sigma_{0}^{2}}}}, $$

where $\bar {y}_{-i} = \frac {1}{n - 1}{\sum }_{j \ne i} y_{j}$ denotes the mean of the observations without the *i* th data point. Consequently, the leave-one-out predictive density is given by a $\mathcal {N}(\mu _{-i},~1~+~\sigma _{-i}^{2})$ distribution which follows from well-known properties of a product of normal distributions. Example 3 considers data sets that convey the maximal possible evidence for $\mathcal {H}_{0}$ by having a sample mean of $\bar {y} = 0$ and a sample variance of $s^{2} = 1$. The Bayesian LOO estimate under $\mathcal {H}_{1}$ is then given by:

21 $$\begin{array}{@{}rcl@{}} \text{elpd}_{\text{loo}}^{\mathcal{H}_{1}} &=& -\frac{n}{2} \log\left( 2 \pi \right) -\frac{n}{2} \log\left( \frac{n + \tfrac{1}{{\sigma_{0}^{2}}}}{n - 1 + \tfrac{1}{{\sigma_{0}^{2}}}}\right)\\ &&- \frac{ \left( n - 1\right) \left( n + \tfrac{1}{{\sigma_{0}^{2}}}\right)}{2 \left( n - 1 + \tfrac{1}{{\sigma_{0}^{2}}}\right)}. \end{array} $$

The difference in the LOO estimates can be written as:

22 $$ {\Delta}\text{elpd}_{\text{loo}}^{\mathcal{H}_{0}, \mathcal{H}_{1}} = \frac{n}{2} \log\left( \frac{n + \tfrac{1}{{\sigma_{0}^{2}}}}{n - 1 + \tfrac{1}{{\sigma_{0}^{2}}}}\right) + \frac{ n - 1}{2 \left( n - 1 + \tfrac{1}{{\sigma_{0}^{2}}}\right)}. $$

As the sample size *n* grows without bound, the difference in the LOO estimates approaches a bound (see Appendix C for a derivation):

23 $$ \lim_{n \to \infty} {\Delta}\text{elpd}_{\text{loo}}^{\mathcal{H}_{0}, \mathcal{H}_{1}} = 1. $$

Consequently, the limit of the pseudo-Bayes factor is

24 $$ \lim_{n \to \infty} \text{PSBF}_{01} = e \approx 2.718, $$

and the limit of the model weight for $\mathcal {H}_{0}$ is

25 $$ \lim_{n \to \infty} w_{0} = \frac{e}{1 + e} \approx 0.731,  $$

which is identical to the limit obtained in example 2.

### Select Scenarios

**Fig. 3:**
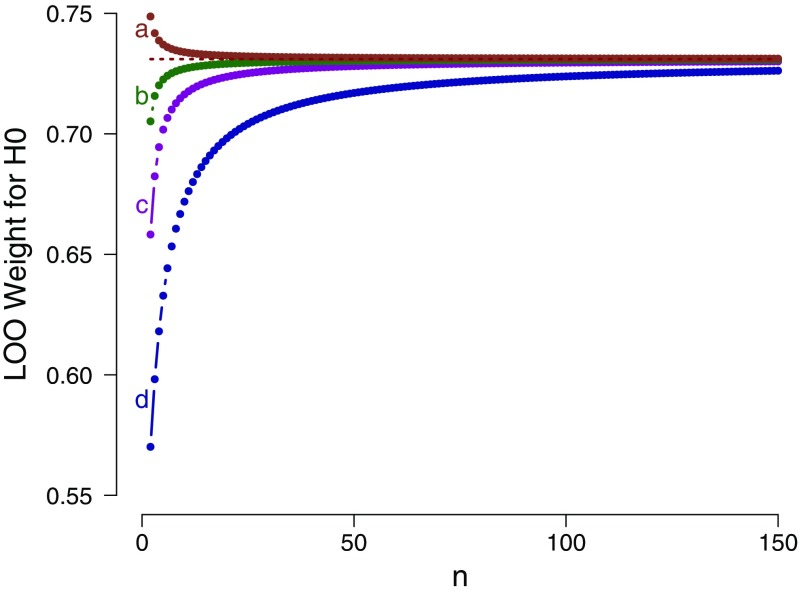
Example 3: LOO weights for $\mathcal {H}_{0}: \mu = 0$ as a function of sample size *n*, for data sets with sample mean equal to zero and sample variance equal to one, evaluated in relation to four different prior specifications for $\mathcal {H}_{1}$: **a** $\mathcal {H}_{1}: \mu \sim \mathcal {N}(0, 3^{2})$; **b** $\mathcal {H}_{1}: \mu \sim \mathcal {N}(0, 1.5^{2})$; **c** $\mathcal {H}_{1}: \mu \sim \mathcal {N}(0, 1)$; and **d** $\mathcal {H}_{1}: \mu \sim \mathcal {N}(0, 0.5^{2})$. The dotted horizontal line indicates the corresponding analytical asymptotic bound (see text for details). Available at https://tinyurl.com/y7qhtp3o under CC license https://creativecommons.org/licenses/by/2.0/

As in the previous two examples, the mathematical result can be applied to a series of select scenarios. Figure 3 shows the LOO weight in favor of the general law $\mathcal {H}_{0}$ as a function of the sample size *n* with sample mean exactly zero and sample variance exactly one, separately for four different prior specifications of $\mathcal {H}_{1}$. The figure confirms that for each prior specification, the LOO weight for $\mathcal {H}_{0}$ approaches the asymptotic bound as *n* grows large.

We conclude the following: (1) as *n* grows large, the support for the general law $\mathcal {H}_{0}$ approaches a bound; (2) in contrast to example 1, but consistent with example 2, this bound is independent of the particular choice of normal prior distribution for $\mu $ under $\mathcal {H}_{1}$; however, consistent with both earlier examples, this bound is surprisingly low. Even with an infinite number of observations and a sample mean of exactly zero, the model weight on the general law $\mathcal {H}_{0}$ does not exceed a modest 0.731; (3) as shown by case **a** in Fig. 3, the choice of a $\mathcal {N}(0,3^{2})$ prior distribution results in a function that approaches the asymptote from above. This means that, according to LOO, increasing the sample size of observations that are perfectly consistent with $\mathcal {H}_{0}$ actually decreases the support for $\mathcal {H}_{0}$, violating the second desideratum outlined earlier; and (4) some prior distributions (e.g., $\mu \sim \mathcal {N}(0,2.035^{2})$) result in a nonmonotonic relation, where the addition of $\mathcal {H}_{0}$-consistent observations initially increases the support for $\mathcal {H}_{0}$, and later decreases it toward asymptote.^6^

In sum, the result of the LOO procedure for a *z* test involving $\mathcal {H}_{0}: \mu = 0$ shows a behavior similar to that for the test of induction ($\mathcal {H}_{0}: \theta = 0$ or $\mathcal {H}_{0}: \theta = 1$) and the test against chance ($\mathcal {H}_{0}: \theta = 1/2$); this behavior violates two seemingly uncontroversial desiderata of inference, namely that the additional observation of data that are perfectly consistent with the general law $\mathcal {H}_{0}$ ought to result in more support for $\mathcal {H}_{0}$, and do so without bound.

## Closing Comments

Three simple examples revealed some expected as well as some unexpected limitations of Bayesian leave-one-out cross-validation or LOO. In the statistical literature, it is already well known that LOO is inconsistent (Shao 1993), meaning that the true data-generating model will not be chosen with certainty as the sample size approaches infinity. Our examples provide a concrete demonstration of this phenomenon; moreover, our examples highlighted that, as the number of $\mathcal {H}_{0}$-consistent observations *n* increases indefinitely, the bound on support in favor of $\mathcal {H}_{0}$ may remain modest. Inconsistency is arguably not a practical problem when the support is bounded at a level of evidence that is astronomically large, say a weight of 0.99999999; however, for both the test against chance and the *z* test, the level of asymptotic LOO support for $\mathcal {H}_{0}$ was categorized by Jeffreys (1939) as “not worth more than a bare comment” (p. 357).

It thus appears that, when the data are generated from a simple model, LOO falls prey to the Scylla of overfitting, giving undue preference to the complex model. The reason for this cuts to the heart of cross-validation: when two candidate models are given access to the same training set, this benefits the complex more than it benefits the simple model. In our examples, the simple model did not have any free parameters at all, and consequently these models gained no benefit whatsoever from having been given access to the training data; in contrast, the more complex models did have free parameters, and these parameters greatly profited from having been given access to the data set. Perhaps this bias may be overcome by introducing a cost function, such that the price for advance information (i.e., the training set) depends on the complexity of the model—models that stand to benefit more from the training set should pay a higher price for being granted access to it. Another approach is to abandon the leave-one-out idea and instead decrease the size of the training set as the number of observations *n* increases;^7^Shao (1993) demonstrated that this approach can yield consistency.

In order to better understand the behavior of leave-one-out cross-validation, it is also useful to consider AIC, a method to which it is asymptotically equivalent (Stone 1977). Indeed, for example 2 and example 3, the asymptotic LOO model weight equals that obtained when using AIC (Burnham and Anderson 2002; Wagenmakers and Farrell 2004). In addition, as pointed out by O’Hagan and Forster (2004, p. 187), “AIC corresponds to a partial Bayes factor in which one-fifth of the data are applied as a training sample and four-fifths are used for model comparison.” O’Hagan and Forster (2004) further note that this method is not consistent. It is also not immediately clear, in general, why setting aside one-fifth of the data for training is a recommendable course of action.

Another unexpected result was that, depending on the prior distribution, adding $\mathcal {H}_{0}$-consistent information may decrease the LOO preference for $\mathcal {H}_{0}$; sometimes, as the $\mathcal {H}_{0}$-consistent observations accumulate, the LOO preference for $\mathcal {H}_{0}$ may even be nonmonotonic, first increasing (or decreasing) and later decreasing (or increasing).

The examples outlined here are simple, and a LOO proponent may argue that, in real-world applications of substantive interest, simple models are never true, that is, the asymptotic data are never fully consistent with a simple model. Nevertheless, when researchers use LOO to compare two different models, it is important to keep in mind that the comparison is not between the predictive adequacy of the two models as originally entertained; the comparison is between predictive adequacy of two models where both have had advance access to all of the observations except one.

In sum, cross-validation is an appealing method for model selection. It directly assesses predictive ability, it is intuitive, and oftentimes it can be implemented with little effort. In the literature, it is occasionally mentioned that a drawback of cross-validation (and specifically LOO) is the computational burden involved. We believe that there is another, more fundamental drawback that deserves attention, namely the fact that LOO violates several common sense desiderata of statistical support. Researchers who use LOO to adjudicate between competing mathematical models for cognition and behavior should be aware of this limitation and perhaps assess the robustness of their LOO conclusions by employing alternative procedures for model selection as well.

## Appendix A: Derivation Example 1—Induction

To investigate how the difference in the LOO estimates

$$\begin{array}{@{}rcl@{}} {\Delta}\text{elpd}_{\text{loo}}^{\mathcal{H}_{0}, \mathcal{H}_{1}} &=& \text{elpd}_{\text{loo}}^{\mathcal{H}_{0}} - \text{elpd}_{\text{loo}}^{\mathcal{H}_{1}} \\ &=& - \log\left[ \left( \frac{a + n - 1}{a + n - 1 + b}\right)^{n} \right] \end{array} $$

behaves as the number of observations goes to infinity, one can consider the limit of $\left (\frac {a + n - 1}{a + n - 1 + b}\right )^{n}$ as $n \to \infty $:

$$\lim_{n \to \infty} \left( \frac{a + n - 1}{a + n - 1 + b}\right)^{n} = \exp\left\{\lim_{n \to \infty} \frac{\log\left[\frac{a + n - 1}{a + n - 1 + b}\right]}{\frac{1}{n}}\right\}. $$

The limit of the denominator is $\lim _{n \to \infty } \frac {1}{n} = 0$ and it is also straightforward to show that $\lim _{n \to \infty } \log \left [\frac {a + n - 1}{a + n - 1 + b}\right ] = 0$. Therefore, both the limit of the numerator and of the denominator are 0 and L’Hôpital’s rule can be applied which yields

$$\begin{array}{@{}rcl@{}} &&\lim_{n \to \infty} \left( \frac{a + n - 1}{a + n - 1 + b}\right)^{n} \\\\ &&= \exp\left\{ - \lim_{n \to \infty} \frac{b }{1+ (2 a - 2 + b) \tfrac{1}{n} + \tfrac{a^{2} - 2 a + ab + 1 - b}{n^{2}}}\right\}. \end{array} $$

Hence,

$$\lim_{n \to \infty} \left( \frac{a + n - 1}{a + n - 1 + b}\right)^{n} =\exp\left\{- b\right\}. $$

Therefore, the difference in the Bayesian LOO estimates ${\Delta }\text {elpd}_{\text {loo}}^{\mathcal {H}_{0}, \mathcal {H}_{1}}$ as $n \to \infty $ is given by:

$$\lim_{n \to \infty} {\Delta}\text{elpd}_{\text{loo}}^{\mathcal{H}_{0}, \mathcal{H}_{1}} = b. $$

## Appendix B: Derivation Example 2—Chance

The difference in the LOO estimates can be written as

$$\begin{array}{@{}rcl@{}} {\Delta}\text{elpd}_{\text{loo}}^{\mathcal{H}_{0}, \mathcal{H}_{1}} &=& \log\left[\left( \frac{a + b + n - 1}{2a + n - 2}\right)^{\frac{n}{2}}\right] \\ &&+~\log\left[\left( \frac{a + b + n - 1}{2b + n - 2}\right)^{\frac{n}{2}}\right]. \end{array} $$

To investigate how this difference behaves as the number of observations goes to infinity, one can consider the limit of $\left (\frac {a + b + n - 1}{2a + n - 2}\right )^{\frac {n}{2}}$ and of $\left (\frac {a + b + n - 1}{2b + n - 2}\right )^{\frac {n}{2}}$ as $n \to \infty $. We first introduce a new variable *m* so that $n = 2 m$, where $m = 1,2,3,\ldots $, which ensures that the number of observation is even, and then consider the limits as $m \to \infty $. The limit of the first expression is given by

$$\lim_{m \to \infty} \left( \frac{a + b + 2m - 1}{2a + 2m - 2}\right)^{m} \!= \exp\left\{\!\lim_{m \to \infty} \frac{\log\left( \frac{a + b + 2m - 1}{2a + 2m - 2}\right)}{\frac{1}{m}}\!\right\}. $$

The limit of the denominator is 0 and it is also straightforward to show that the limit of the numerator is 0. Hence, L’Hôpital’s rule can be applied which yields

$$\lim_{m \to \infty} \left( \frac{a + b + 2m - 1}{2a + 2m - 2}\right)^{m} = \exp\left\{\frac{b - a + 1}{2}\right\}. $$

Next, we consider the limit of the expressions in the second logarithm as $m~\to ~\infty $:

$$\lim_{m \to \infty} \left( \!\frac{a + b +\! 2m -\! 1}{2b + 2m -\! 2}\!\right)^{m} = \exp\left\{\!\lim_{m \to \infty} \frac{\log\left( \frac{a + b + 2m - 1}{2b + 2m - 2}\right)}{\frac{1}{m}}\!\right\}. $$

The limit of the denominator is 0 and it is also straightforward to show that the limit of the numerator is 0. Hence, L’Hôpital’s rule can be applied which yields

$$\lim_{m \to \infty} \left( \frac{a + b + 2m - 1}{2b + 2m - 2}\right)^{m} = \exp\left\{\frac{a - b + 1}{2}\right\}. $$

Therefore, the difference in the LOO of the two models as $m \to \infty $ is given by:

$$\begin{array}{@{}rcl@{}} \lim_{m \to \infty} \left[{\Delta}\text{elpd}_{\text{loo}}^{\mathcal{H}_{0}, \mathcal{H}_{1}}\right] &=& \frac{b - a + 1}{2} + \frac{a - b + 1}{2} \\ &=& 1. \end{array} $$

## Appendix C: Derivation Example 3—Nullity of a Normal Mean

We first show how to obtain the expression for the difference in the LOO estimates. Note that the LOO estimate under $\mathcal {H}_{1}$ can be written as:

$$\begin{array}{@{}rcl@{}} \text{elpd}_{\text{loo}}^{\mathcal{H}_{1}} = &-&\frac{n}{2} \log\left( 2 \pi \right) -\frac{n}{2} \log\left( \frac{n + \frac{1}{{\sigma_{0}^{2}}}}{n - 1 + \frac{1}{{\sigma_{0}^{2}}}}\right)\\ &-& \frac{n - 1 + \frac{1}{{\sigma_{0}^{2}}}}{2 \left( n + \frac{1}{{\sigma_{0}^{2}}}\right)} \sum\limits_{i = 1}^{n} {y_{i}^{2}} \\ &+& \frac{n - 1}{n + \frac{1}{{\sigma_{0}^{2}}}} \sum\limits_{i = 1}^{n} y_{i} \bar{y}_{-i} \\ &-& \frac{(n - 1)^{2}}{2 \left( n + \frac{1}{{\sigma_{0}^{2}}}\right) \left( n - 1 + \frac{1}{{\sigma_{0}^{2}}}\right)} \sum\limits_{i = 1}^{n} \bar{y}_{-i}^{2}. \end{array} $$

Since we consider data sets that have a sample mean of exactly 0, we know that ${\sum }_{i = 1}^{n} y_{i} = 0$ so that ${\sum }_{j \ne i} y_{j} = -y_{i}$. Furthermore, since the sample variance is exactly 1 and the sample mean is exactly zero, we know that $s^{2} = 1 = \frac {1}{n - 1} {\sum }_{i = 1}^{n} (y_{i} - 0)^{2}$, hence, ${\sum }_{i = 1}^{n} {y_{i}^{2}} = n - 1$. Using these observations, one can show that

$$\begin{array}{@{}rcl@{}} \sum\limits_{i = 1}^{n} y_{i} \bar{y}_{-i} &=& \sum\limits_{i = 1}^{n} y_{i} \left[\frac{1}{n - 1} \sum\limits_{j \ne i} y_{j}\right] \\ &=& \sum\limits_{i = 1}^{n} y_{i} \left[- \frac{1}{n - 1} y_{i}\right] \\ &=& - \frac{1}{n - 1} \underbrace{\sum\limits_{i = 1}^{n} {y_{i}^{2}}}_{n - 1} \\ &=& -1, \end{array} $$

and

$$\begin{array}{@{}rcl@{}} \sum\limits_{i = 1}^{n} \bar{y}_{-i}^{2} &= \sum\limits_{i = 1}^{n} \left[- \frac{1}{n - 1} y_{i}\right]^{2} \\ &= \frac{1}{\left( n - 1\right)^{2}} \underbrace{\sum\limits_{i = 1}^{n} {y_{i}^{2}}}_{n - 1} \\ & = \frac{1}{n - 1}. \end{array} $$

Hence, using these results and after some further simplifications, the LOO estimate under $\mathcal {H}_{1}$ can be written as:

$$\begin{array}{@{}rcl@{}} \text{elpd}_{\text{loo}}^{\mathcal{H}_{1}} = &-&\frac{n}{2} \log\left( 2 \pi \right) -\frac{n}{2} \log\left( \frac{n + \frac{1}{{\sigma_{0}^{2}}}}{n - 1 + \frac{1}{{\sigma_{0}^{2}}}}\right)\\ &-& \frac{ \left( n - 1\right) \left( n + \frac{1}{{\sigma_{0}^{2}}}\right)}{2 \left( n - 1 + \frac{1}{{\sigma_{0}^{2}}}\right)}. \end{array} $$

Therefore, the difference in the LOO estimates can be written as:

$${\Delta}\text{elpd}_{\text{loo}}^{\mathcal{H}_{0}, \mathcal{H}_{1}} = \log\left[\left( \frac{n + \frac{1}{{\sigma_{0}^{2}}}}{n - 1 + \frac{1}{{\sigma_{0}^{2}}}}\right)^{\frac{n}{2}}\right] + \frac{ n - 1}{2 \left( n - 1 + \frac{1}{{\sigma_{0}^{2}}}\right)}. $$

To investigate how this difference behaves as the number of observations goes to infinity, we take the limit of each of the terms. The limit of the first term is obtained by taking the limit of the expression in the logarithm:

$$\lim_{n \to \infty} \left( \frac{n + \frac{1}{{\sigma_{0}^{2}}}}{n - 1 + \frac{1}{{\sigma_{0}^{2}}}}\right)^{\frac{n}{2}} = \exp\left\{\frac{1}{2} \lim_{n \to \infty} \frac{\log\left[\frac{n + \frac{1}{{\sigma_{0}^{2}}}}{n - 1 + \frac{1}{{\sigma_{0}^{2}}}}\right]}{\frac{1}{n}}\right\}. $$

The limit of the denominator is 0 and it is also straightforward to show that the limit of the numerator is 0. Hence, L’Hôpital’s rule can be applied which yields

$$\lim_{n \to \infty} \left( \tfrac{n + \tfrac{1}{{\sigma_{0}^{2}}}}{n - 1 + \tfrac{1}{{\sigma_{0}^{2}}}}\right)^{\frac{n}{2}} = \exp\left\{\frac{1}{2}\right\}. $$

The limit of the second term is given by:

$$\lim_{n \to \infty} \frac{ n - 1}{2 \left( n - 1 + \frac{1}{{\sigma_{0}^{2}}}\right)} = \frac{1}{2}. $$

Therefore, the difference in the LOO of the two models as $n \to \infty $ is given by:

$$\begin{array}{@{}rcl@{}} \lim_{n \to \infty} \left[{\Delta}\text{elpd}_{\text{loo}}^{\mathcal{H}_{0}, \mathcal{H}_{1}}\right] &=& \log\left[\exp\left\{\frac{1}{2}\right\}\right] + \frac{1}{2} \\ &=& 1. \end{array} $$

## Footnotes

1 The LOO functionality is available through the R package “loo” (Vehtari et al. 2018), see also http://mc-stan.org/loo/.

2 “[...] it is known to many statisticians (although a rigorous statement has probably not been given in the literature) that the cross-validation with $n_{v} \equiv 1$ is asymptotically incorrect (inconsistent) and is too conservative in the sense that it tends to select an unnecessarily large model” (Shao 1993, p. 486).

3 The authors agree with Bayarri et al. (2012, p. 1553) who argued that “[...] it would be philosophically troubling to be in a situation with infinite data generated from one of the models being considered, and not choosing the correct model.”

4 Note that the following expressions are conditional on a specific model. However, we have omitted conditioning on the model for enhanced legibility.

5 This holds for *k*-fold CV in general.

6 Because the size of this nonmonotonicity is relatively small, we have omitted it from the figure. The OSF project page https://osf.io/6s5zp/ contains a figure that zooms in on the nonmonotonicity.

7 Critics of cross-validation might argue that one weakness of the approach is that it is not a unique method for assessing predictive performance. That is, users of cross-validation need to decide which form to use exactly (e.g., leave-one-out, leave-two-out, *k*-fold), and different choices generally yield different results.

## References

[CR1] Akaike, H. (1973). Information theory as an extension of the maximum likelihood principle. In Petrov, B.N., & Csaki, F. (Eds.) *2nd international symposium on information theory* (pp. 267–281). Budapest: Akademiai Kiado.

[CR2] Ando T (2010). Bayesian model selection and statistical modeling.

[CR3] Bayarri MJ, Berger JO, Forte A, García-Donato G (2012). Criteria for Bayesian model choice with application to variable selection. The Annals of Statistics.

[CR4] Bem DJ (2011). Feeling the future: Experimental evidence for anomalous retroactive influences on cognition and affect. Journal of Personality and Social Psychology.

[CR5] Browne M (2000). Cross-validation methods. Journal of Mathematical Psychology.

[CR6] Burnham KP, Anderson DR (2002). Model selection and multimodel inference: A practical information–theoretic approach.

[CR7] Claeskens G, Hjort NL (2008). Model selection and model averaging.

[CR8] Doxas I, Dennis S, Oliver WL (2010). The dimensionality of discourse. Proceedings of the National Academy of Sciences.

[CR9] Etz A, Wagenmakers E-J (2017). J. B. S. Haldane’s contribution to the Bayes factor hypothesis test. Statistical Science.

[CR10] Geisser S (1975). The predictive sample reuse method with applications. Journal of the American Statistical Association.

[CR11] Geisser S, Eddy WF (1979). A predictive approach to model selection. Journal of the American Statistical Association.

[CR12] Gelfand AE, Dey DK (1994). Bayesian model choice: Asymptotics and exact calculations. Journal of the Royal Statistical Society. Series B (Methodological).

[CR13] Gelfand, A.E., Dey, D.K., Chang, H. (1992). Model determination using predictive distributions with implementation via sampling-based methods. In Bernardo, J.M., Berger, J.O., Dawid, A.P., Smith, A.F.M. (Eds.) *Bayesian statistics 4* (pp. 147–167). Oxford: Oxford University Press.

[CR14] Gelman A, Hwang J, Vehtari A (2014). Understanding predictive information criteria for Bayesian models. Statistics and Computing.

[CR15] Gronau, Q.F., & Wagenmakers, E.-J. (in press). Bayesian evidence accumulation in experimental mathematics: A case study of four irrational numbers. *Experimental Mathematics*.

[CR16] Grünwald P (2007). The minimum description length principle.

[CR17] Grünwald, P., Myung, I. J., Pitt, M. A. (Eds.). (2005). *Advances in minimum description length: Theory and applications*. Cambridge: MIT Press.

[CR18] Hastie T, Tibshirani R, Friedman J, Vetterling W (2008). The elements of statistical learning.

[CR19] Jeffreys H (1939). Theory of probability.

[CR20] Jeffreys H (1961). Theory of probability.

[CR21] Kass RE, Raftery AE (1995). Bayes factors. Journal of the American Statistical Association.

[CR22] Lee MD, Vanpaemel W (2018). Determining informative priors for cognitive models. Psychonomic Bulletin & Review.

[CR23] Lindley DV (1985). Making decisions.

[CR24] Ly A, Verhagen AJ, Wagenmakers E-J (2016). Harold Jeffreys’s default Bayes factor hypothesis tests: Explanation, extension, and application in psychology. Journal of Mathematical Psychology.

[CR25] Mulder J, Wagenmakers E-J (2016). Editor’s introduction to the special issue on “Bayes factors for testing hypotheses in psychological research: Practical relevance and new developments”. Journal of Mathematical Psychology.

[CR26] Myung IJ (2000). The importance of complexity in model selection. Journal of Mathematical Psychology.

[CR27] Myung IJ, Forster MR, Browne MW (2000). Model selection [Special issue]. Journal of Mathematical Psychology.

[CR28] Myung IJ, Navarro DJ, Pitt MA (2006). Model selection by normalized maximum likelihood. Journal of Mathematical Psychology.

[CR29] Myung IJ, Pitt MA (1997). Applying Occam’s razor in modeling cognition: A Bayesian approach. Psychonomic Bulletin & Review.

[CR30] Nathoo FS, Masson MEJ (2016). Bayesian alternatives to null–hypothesis significance testing for repeated–measures designs. Journal of Mathematical Psychology.

[CR31] O’Hagan A, Forster J (2004). Kendall’s advanced theory of statistics vol 2B: Bayesian inference.

[CR32] Rissanen J (2007). Information and complexity in statistical modeling.

[CR33] Rouder JN, Morey RD, Speckman PL, Province JM (2012). Default Bayes factors for ANOVA designs. Journal of Mathematical Psychology.

[CR34] Schwarz G (1978). Estimating the dimension of a model. Annals of Statistics.

[CR35] Shao J (1993). Linear model selection by cross–validation. Journal of the American Statistical Association.

[CR36] Stone M (1974). Cross–validatory choice and assessment of statistical predictions (with discussion). Journal of the Royal Statistical Society B.

[CR37] Stone M (1977). An asymptotic equivalence of choice of model by cross-validation and Akaike’s criterion. Journal of the Royal Statistical Society Series B.

[CR38] Vandekerckhove, J., Matzke, D., Wagenmakers, E.-J. (2015). Model comparison and the principle of parsimony. In Busemeyer, J., Townsend, J., Wang, Z.J., Eidels, A. (Eds.) *Oxford handbook of computational and mathematical psychology* (pp. 300–319). Oxford: Oxford University Press.

[CR39] Vehtari, A., Gabry, J., Yao, Y., Gelman, A. (2018). loo: Efficient leave-one-out cross-validation and WAIC for Bayesian models. Retrieved from https://CRAN.R-project.org/package=loo(Rpackageversion2.0.0)https://CRAN.R-project.org/package=loo(Rpackageversion2.0.0).

[CR40] Vehtari A, Gelman A, Gabry J (2017). Practical Bayesian model evaluation using leave-one-out cross-validation and WAIC. Statistics and Computing.

[CR41] Vehtari A, Ojanen J (2012). A survey of Bayesian predictive methods for model assessment, selection and comparison. Statistics Surveys.

[CR42] Wagenmakers E-J, Farrell S (2004). AIC model selection using Akaike weights. Psychonomic Bulletin & Review.

[CR43] Wagenmakers, E. -J., & Waldorp, L. (2006). Model selection: Theoretical developments and applications [Special issue]. *Journal of Mathematical Psychology 50*(2).

[CR44] Wrinch D, Jeffreys H (1921). On certain fundamental principles of scientific inquiry. Philosophical Magazine.

[CR45] Yao, Y., Vehtari, A., Simpson, D., Gelman, A. (in press). Using stacking to average Bayesian predictive distributions. *Bayesian Analysis*.

[CR46] Yarkoni T, Westfall J (2017). Choosing prediction over explanation in psychology: Lessons from machine learning. Perspectives on Psychological Science.

